# Developmental stage on day-5 and fragmentation rate on day-3 can influence the implantation potential of top-quality blastocysts in IVF cycles with single embryo transfer

**DOI:** 10.1186/1477-7827-5-2

**Published:** 2007-01-26

**Authors:** Tiziana della Ragione, Greta Verheyen, Evangelos G Papanikolaou, Lisbet Van Landuyt, Paul Devroey, Andre Van Steirteghem

**Affiliations:** 1 Centre for Reproductive Medicine, University Hospital, Vrije Universiteit Brussel, Laarbeeklaan 101, B-1090 Brussels, Belgium

## Abstract

**Background:**

In IVF-ICSI cycles with single embryo transfer (SET), embryo selection for transfer is of crucial importance. The present study aimed to define which embryo parameters might be related to the implantation potential of advanced blastocysts.

**Methods:**

Overall, in 203 cycles with SET, developmental characteristics of 93 implanted (group A) and 110 non-implanted (group B) advanced blastocysts of good quality were compared. The following developmental parameters were assessed in the two groups: normal fertilization, developmental stage on day 5, number of blastomeres on day 2 and on day 3, fragmentation rate on day 3, compaction on day 4 and cleavage pattern on day 2 and day 3.

**Results:**

Expanded blastocysts compared to full blastocysts have higher implantation potential (56.5% vs. 29.3%, p < 0.05). In group B, a higher proportion of advanced blastocysts showed between 10% and 50% anucleated fragments on day 3 than in group A (23.6 vs 11.8, P = 0.03). Advanced blastocysts with >10–50% fragments on day 3 showed a significant lower implantation (29.7%) than those with ≤ 10%fragments (49.4%, P = 0.03). All the other parameters analysed were comparable for the two groups.

**Conclusion:**

Developmental stage on day 5 and fragmentation rate on day 3 were related to the implantation potential of advanced blastocysts and should also be taken into account in the selection of the best advanced blastocyst for transfer.

## Background

The ultimate goal of assisted procreation is the birth of a singleton liveborn infant. Growing concerns have been raised regarding the risks of multiple pregnancies which consist of approximately 30% of the deliveries after assisted reproductive technology (ART) [1]. The only way to achieve this goal is to move towards single embryo transfer policy [2]. In order to prevent a significant decrease in pregnancy and delivery rates when only one embryo is transferred, the selection of that embryo for transfer should be optimal to ensure a high implantation rate.

There is general agreement that a positive correlation exists between embryo quality and pregnancy rates [3]. Several studies, either on day 2 [4-9] have attempted to quantify the implantation potential of an embryo by means of scoring systems based on different embryological parameters such as blastomere size, cell number, cleavage speed, degree of fragmentation. These factors have been combined in a variety of different ways to yield embryo-scoring systems that could predict pregnancy [10,11]. Van Royen et al. reported an interesting approach, in which they estimated the implantation potential of day 3 embryos, comparing all the embryos leading to an ongoing implantation to those that did not result in a pregnancy [12].

On the other hand, the feasibility of culturing the human embryo to a viable blastocyst, using appropriate sequential media, allows for the identification of those embryos with a higher developmental potential [13]. Furthermore, to perform embryo transfer at the blastocyst stage has additional advantages such as better synchronization between embryo developmental stage and uterine environment [14], and decreased uterine contractility [15]. However, by extending the embryo culture to day-5 there is a risk that the embryos of some patients could not reach that stage and consequently the cancellation of transfer will deprive them from a chance of pregnancy. Nevertheless, controversy still exists in literature regarding the merits of blastocyst transfer [16,17].

So far, no attempt has been made to correlate specific embryo parameters of blastocysts and their implantation potential. Moreover, single embryo transfer offers the possibility of comparing two embryo populations of implanted and non-implanted embryos, thus eliminating biases by excluding transfers where only one embryo implanted. This was the case in previous studies based on double embryo transfers (DET) [12].

The aim of the present retrospective study was to assess whether day2 – day3 – day4, and day5 embryo parameters of selected advanced blastocysts are related to their implantation potential. In cycles with SET on day 5, developmental characteristics of implanted and non-implanted blastocysts were compared. This may provide evidence on the selection criteria of the advanced blastocyst with the highest implantation potential in cases where more than one advanced blastocyst is available on the day of transfer.

## Methods

### Study design

Between January 2001 and March 2004, 312 single embryo transfers were performed on day 5 in patients under 36 years old in their first or second IVF attempt. Cycles with preimplantation genetic diagnosis (PGD), frozen embryo transfer cycles and cycles with frozen or non-ejaculated sperm were not included.

In order to study which embryo characteristics – observed during the culture period preceding the blastocyst transfer and on the fifth day of the embryo culture – may interfere with the implantation potential of the blastocyst, we reduced the variability in quality of the transferred blastocysts and we included in the study only good quality blastocysts. Therefore, only the advanced blastocysts of good quality (BL3 and BL4 according to Gardner and Schoolcraft), [18], with type A or B of inner cell mass and for trophectoderm (n = 203, 65.1%), were considered for the study [18]. Ninety-three implanting (group A) and one hundred and ten non-implanting blastocysts (group B) were compared for the analysis.

Transfer with compacted embryos (n = 25, 8%), with early blastocysts or with advanced blastocysts of inferior quality (n = 84, 26.9%) were excluded.

### Assisted reproduction techniques

Female patients underwent multifollicular ovarian stimulation (multi-FOS) with a GnRH antagonist/recombinant gonadotrophin protocol. The details about stimulation protocol have been published elsewhere [19]. When three or more follicles reached a size of ≥ 17 mm in diameter, final oocyte maturation was induced by the administration of 10 000 IU human chorionic gonadotrophin (hCG). Oocyte retrieval was carried out 36 h later by vaginal ultrasound-guided puncture of ovarian follicles.

Standard IVF/ICSI procedures were applied according to Van Landuyt et al. [20]. For the IVF procedure, each cumulus-oocyte complex was placed in a 25 μl droplet of fertilization medium covered by lightweight paraffin oil. Cumulus-oocyte complexes were inseminated within 3–4 hours after retrieval by adding 5000 progressively motile spermatozoa (type A + B motility).

For the ICSI procedure, denudation of the cumulus cells was carried out by exposure to 10 IU of hyaluronidase (type VIII, Sigma Chemical Company, St. Louis, Mo) [21]. Nuclear maturation of the oocytes was checked after denudation. Only mature, metaphase II oocytes were used for ICSI as described previously [22]. Injected oocytes were placed in 25 μl droplets of cleavage medium and incubated under an atmosphere of 6% CO_2_, 5% O_2 _and 89% N_2 _at 37°C. Two different sequential media were used for oocyte and embryo culture (GII series from Vitrolife, Göteborg, Sweden or BlastAssist System from MediCult, Jyllinge, Denmark).

### Evaluation of fertilization and embryo quality

Survival and fertilization of the oocytes were checked 16–19 h after injection using an inverted microscope at 400× magnification. The zygotes were scored taking into account number, position and size of the pronuclei as well as number and location of the nucleolar precursor bodies within the pronuclei. Normal fertilization was confirmed by the presence of two pronuclei (PN) with two distinct or fragmented polar bodies. Fertilization rate was expressed as the percentage of 2PN oocytes or the number of inseminated (IVF) or injected (ICSI) oocytes. Embryo development and embryo quality were assessed daily until the moment of intrauterine transfer. Embryo quality was scored according to the following criteria: the number of blastomeres, the rate of fragmentation, cell-size symmetry, multinucleation, the degree of compaction, granulation and the presence of vacuoles. We considered five categories of fragmentation: type 0 embryos had no anucleated fragments, type 1 embryos showed >0% – ≤10% fragments, type 2 embryos showed >10% – ≤20% fragments, type 3 embryos showed >20% – ≤50% fragments and type 4 embryos showed >50% fragments (type 4 was not transferred). Day-2 and Day-3 evaluations were carried out 43–46 hours and 65–68 hours after insemination/injection respectively. In the morning of day 3, embryos were rinsed and transferred to individual 25 μl fresh blastocyst medium droplets covered by paraffin oil.

### Embryo parameters analysed for implanted and non-implanted advanced blastocysts

The two groups were compared for the following developmental parameters: number of blastomeres on day 2 (<4 cells, 4 cells, >4 cells) and on day 3 (<8 cells, 8 cells, >8 cells); fragmentation rate on day 3 (≤10% and >10–50%); compaction on day 4 (non-compacting, compacting + compacted, early blastocyst), and cleavage pattern. The latter was evaluated in two different ways. First, the cleavage stage on day 3 (<8 cells, 8 cells, > 8 cells, other pattern) of the only embryos that had 4 cells on day 2 was investigated. Secondly, the cleavage stage on day 2 (<4 cells, 4 cells, > 4 cells) of the only embryos that had 8 cells on day 3 was evaluated.

### Evaluation of blastocyst quality

Blastocyst evaluation was performed on day 5 of in-vitro culture according to the Gardner and Schoolcraft criteria [19]. This blastocyst scoring system is based on three parameters: (i) blastocoel formation and degree of expansion, (ii) development of the inner cell mass (ICM) and (iii) development of the trophectoderm (TE). Only advanced blastocysts (type 3 full blastocysts and type 4 expanded blastocysts) with a good quality inner cell mass (many cells tightly packed type A or several cells loosely grouped type B) and multicellular trophectoderm (many cells forming a cohesive epithelium type A or few cells forming a loose epithelium type B) were included in our study (BL3AA, BL3AB, BL3BA, BL3BB, BL4AA, BL4AB, BL4BA, BL4BB). On the morning of day 5, the embryologist selected the "best" blastocyst for transfer. This choice was based on several criteria. Preferably a full or an advanced blastocyst was selected, with a clear inner cell mass and several cells in the trophectoderm. If none of the embryos fulfilled these criteria, early blastocysts or compacted embryos were transferred.

### Embryo transfer and pregnancy

Embryo transfer was carried out on day 5. In the present study, only SETs were taken into consideration. The first pregnancy test was performed 14 days after the oocyte retrieval. A pregnancy was confirmed when two consecutive hCG concentrations of >10 IU/L were measured. Clinical pregnancy was defined by intrauterine gestational sac with fetal heartbeat, observed by vaginal ultrasound at 7 weeks of gestation. The implantation rate was considered as the percentage of fetal sacs with heartbeat on the number of embryos transferred.

### Statistical analysis

Fisher's exact test was used to analyse nominal variables (fragmentation rate on day 3, compaction degree on day 4 and cleavage pattern, number of blastomeres on day 2 and on day 3, type of insemination procedure) in the form of frequency tables. Normally distributed (Kolmogorov-Smirnov Test with Lilliefors correction) metric variables (gonadotrophins total dose, number of oocytes, GV, MI, MII and 2PN) were tested with the t-test for independent samples, while non-normally distributed metric variables (age of patients) were analysed with the Mann-Whitney U test. All tests were two-tailed with a confidence level of 95% (p < 0.05). Values are expressed as mean ± standard error of the mean (SEM). Regression analysis performed where appropriate.

## Results

Overall 203 advanced blastocysts were considered. The characteristics of two embryo populations were compared: 93 implanted embryos (group A) and 110 non-implanted embryos (group B). The implantation rate was 45.8%.

Comparing group A with group B, there was no difference with regard to the patients' age (29.7 ± 0.3 vs. 30.1 ± 0.3), the total dose of gonadotrophins used (1633 ± 69 vs. 1720 ± 73) and the type of insemination procedure performed: ICSI (66.7% vs. 70.0%), IVF (31.2% vs. 26.4%) and ICSI in combination with IVF (2.2% vs. 3.6%). The two groups were also comparable with relation to the mean number of COCs retrieved and the mean percentages of GV, MI oocytes and MII oocytes per cycle. Furthermore, similar results were found with regard to normal fertilization rate (%2PN) (Table 1).

**Table 1:** Patient and cycle characteristics

	**Group A (100% implantation)**	**Group B (O% implantation)**	**Statistics**
**No. of Patients (embryos)**	93	110	
**Age**^1^	29.7 ± 0.3	30.1 ± 0.3	NS
**Gonadotrophins total dose**^1^	1633 ± 69	1720 ± 73	NS
**Type of insemination procedure No. (%)**			
∘ **ICSI**	62 (66.7)	78 (71.0)	NS
∘ **IVF**	31 (33.3)	32 (29.0)	
**No. of oocytes**^1^	13.6 ± 0.7	13.0 ± 0.6	NS
**No. of GV**^2^	1.0(6.8)	0.9 (6.4)	NS
**No. of MI**^2^	0.2 (1.7)	0.3 (2.3)	NS
**No. of MII**^2^	12.0 (89.8)	11.0 (86.1)	NS
**No. of 2PN**^2^	8.2 (64.1)	7.5 (58.9)	NS
**Blastulation rate%**	55%	49%	NS

SEM = standard error of the mean

NS = not significant

^1^Values expressed as mean ± SEM

^2^Values expressed as mean number (%)

The individual implantation potential of each of the eight categories of advanced blastocysts is shown in Table 2. Comparing expanded blastocysts (BL4) to full blastocysts (BL3) (Table 2), it seems that BL4 stage has better pregnancy outcome than BL3 stage, and certainly a top quality BL4 embryo (BL4AA & BL4AB) has a higher implantation potential than a top quality BL3 embryo (BL3AA & BL3AB) (52.4% vs. 28.3%, p < 0.05, respectively).

**Table 2:** Effect of blastocyst score on pregnancy outcome

**Different categories of advanced blastocysts**	**Not implanted % (n)**	**Implanted % (n)**	**P value**
**Total BL3 (n = 81) **(*BL3AA, BL3AB, BL3BA, BL3BB*)	70.3% (57)	29.3%(24)	
**Total BL4 (n = 122) **(*BL4AA, BL4AB, BL4BA, BL4BB*)	43.4% (53)	56.5%(69)	p < 0.05
**Top quality BL3 (n = 76) **(*BL3AA, BL3AB*)	65.4% (53)	28.3%(23)	
**Top quality BL4 (n = 109) **(*BL4AA, BL4AB*)	36.8% (45)	52.4%(64)	p < 0.05
**Individual implantation rate of advanced blastocysts**			
• **BL3AA (n = 62)**	67.7% (42)	32.3% (20)	
• **BL3AB (n = 14)**	78.6% (11)	21.4% (3)	
• **BL3BA (n = 2)**	50% (1)	50% (1)	
• **BL3BB (n = 3)**	100% (3)	0	P = 0.02
• **BL4AA (n = 107)**	43% (46)	57% (61)	
• **BL4AB (n = 4)**	25% (1)	75% (3)	
• **BL4BA (n = 6)**	33.3% (2)	66.7% (4)	
• **BL4BB (n = 5)**	80% (4)	20% (1)	

**Figure 1:**
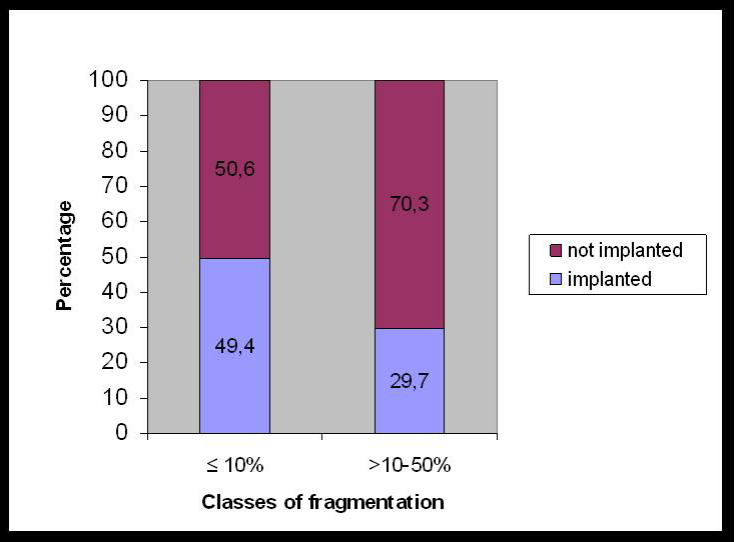
Implantation potential of advanced blastocysts with respect to fragmentation rate on day 3 (≤10% vs. >10–50%).

The comparison of several embryo parameters revealed that the fragmentation rate on day 3 was significantly different for groups A and B (Table 2). The proportion of advanced blastocysts with >10% fragmentation on day 3 was significantly higher (P = 0.03) in group B than in group A (23.6% vs 11.8%). As a result, advanced blastocysts with >10% fragments on day 3 showed a significantly (P = 0.03) lower implantation rate than those with ≤10% fragments (29.7% vs 49.4%) Figure 1.

No differences were observed in the number of blastomeres on day 2 and on day 3, and in the degree of compaction between the implanted and non-implanted blastocysts (Table 3). The proportion of the three embryo stage categories on day 2 (<4 cells, 4 cells, >4 cells), on day 3 (<8 cells, 8 cells, > 8 cells) and on day 4 (non-compacting, compacting + compacted, early blastocyst) was similar for group A and for group B. Similar results were found for the cleavage patterns. From all the 4-cell embryos on day 2, the proportion that reached < 8 cells, 8 cells and > 8 cells on day 3 was comparable for the implanted and non-implanted embryos. From the 8-cell embryos on day 3, almost all of them showed 4 cells on day 2, both in group A and in group B. The proportion of advanced blastocysts that showed 4 blastomeres on day 2 and 8 blastomeres on day 3 was the same in the implanted and non-implanted populations (72.5% group A vs 66.3% group B) (Table 3).

**Table 3:** Embryo parameters analysed in the implanted (Group A) and non-implanted (Group B) advanced blastocysts

	**Group A n = 93**	**Group B n = 110**	**Statistics**
**Number of blastomeres on day 2**			
∘ < 4 cells	2 (2.2%)	4 (3.6%)	NS
∘ 4 cells	80 (86.0%)	95 (86.4%)	
∘ > 4 cells	11 (11.8%)	11 (10.0%)	

**Number of blastomeres on day 3**			
∘ <8 cells	3 (3.2%)	7 (6.4%)	NS
∘ 8 cells	63 (67.7%)	67 (60.9%)	
∘ >8 cells	27 (29.0%)	36 (32.7%)	

**Fragmentation rate on day 3**			
∘ <10%	82 (88.2%)	84 (76.4%)	0.03
∘ ≥ 10–50%	11 (11.8%)	26 (23.6%)	

**Embryo stage on day 4**			
∘ Not compacted	3 (3.2%)	4 (3.6%)	NS
∘ Compacted	68 (73.1%)	73 (66.4%)	
∘ Early blastocysts	22 (23.7%)	33 (30.0%)	

**Cleavage pattern: **4 cells on day 2 vs. number of cells on day 3	n = 80	n = 95	
∘ 4 cells – < 8 cells	3 (3.7%)	5 (5.2%)	NS
∘ 4 cells – 8 cells	58 (72.5%)	63 (66.3%)	
∘ 4 cells – > 8 cells	19 (23.7%)	27 (28.4%)	

**Cleavage pattern**: 8 cells on day 3 vs. number of cells on day 2	n = 63	n = 67	
∘ 8 cells – < 4 cells	2 (3.2%)	2 (3.0%)	NS
∘ 8 cells 4	58 (92.1%)	63 (94%)	
∘ 8 cells – > 4 cells	3 (4.8%)	2 (3.0%)	

Multivariate logistic regression analysis was performed with dependent variable the achievement of implantation or not and independent variables the fragmentation rate on day-3, the developmental stage on day-5 (BL3 or BL4) and the age. The only covariate that was statistically significant was the type of advanced blastocyst on the fifth day of embryo culture (p = 0.032).

A logistic regression analysis failed to find any statistically significant effect of the two sequential media on pregnancy occurrence (data not shown).

## Discussion

The selection of embryos with a high chance of implantation is a difficult task in human assisted reproduction. One of the proposed methods is to extend the culture period up to the blastocyst stage. On day 5, the morphological criteria for selection, although still subjective [23], eliminate embryos with impaired development beyond the 8-cell stage and reduce the risk of transferring a top-quality embryo with chromosomal abnormalities [24]. Furthermore, with the increased application of single embryo transfer, the selection of the "best" embryo has become an urgent need.

To the best of our knowledge, this is the first study which has attempted to assess whether embryo characteristics of advanced good quality blastocysts are related to their implantation potential in a single embryo setting. Performing the study on only SET's eliminates the bias involved in comparing populations with more than one implanted embryo or more than one non-implanted embryo from the same patient.

Our findings suggest that the developmental stage of an advanced blastocyst quantitated by a systematic scoring system, affects its implantation potential, resulting in better pregnancy outcome with an advanced blastocyst stage BL4 (AA or AB) than a full blastocyst BL3 (AA or AB), although all these embryos are characterised as top-quality embryos. The above finding confirms previous reports where patients with expanded blastocysts had higher clinical pregnancy and live birth rates compared with patients with non-expanding blastocysts (morulae and early blastocysts) [25]. It seems rational that we need to re-evaluate our quality ranking according to the grading score for advanced blastocysts since the real expanded blastocysts with a good quality inner cell mass and multicellular trophectoderm (BL4AA and BL4AB) represent a subcategory within the advanced blastocysts with really optimum quality as they can achieve implantation rates as high as 52.4%. Similarly, Gardner et al have found implantation rates as high as 69% by transferring two blastocysts with score ≥3AA, resulting in the suggestion that in the presence of two top-scoring blastocysts, single embryo transfer should be implemented in order to avoid multiple pregnancies [26]. The current study confirms the above proposal and furthermore highlights the higher efficacy of an expanded blastocyst compared to a full blastocyst.

In addition, we observed that the fragmentation rate on day 3 was related to the implantation potential of the advanced blastocysts selected for transfer on day 5. As a result, blastocysts with >10% fragments on day 3 showed a significantly lower implantation rate than those with ≤10% fragments (29.7% vs 49.4%) (Figure 1). Although this significance disappears in the multivariate analysis, that result suggests that fragmentation on day 3 should be more taken into account in the selection of the best blastocyst for transfer in patients where more than one advanced blastocyst of good quality is available on the day of transfer. If only one advanced blastocyst with fragmentation is available, it remains unclear whether this embryo should be selected in preference to an early blastocyst with minimal fragmentation. However, this issue is beyond the scope of the present study.

Eighty percent of the transferred advanced blastocysts in the present study had less than 10% fragmentation on day 3. This seems logical as blastocyst formation decreases with increasing fragmentation rate [27,28]. Although embryos with > 10% fragmentation may also reach the advanced blastocyst stage, their fragmentation rate still had a negative impact on implantation. Since the fragmentation rate does not decrease during development [29], the impact of this parameter was only taken into consideration on day 3. It is well known that a significant decrease in implantation rate and pregnancy rate occur with increased fragmentation, especially between 10–50%, both on day 2 and day 3 [27,28,30]. Cytogenetic data have shown that 47% of highly fragmented (20–40% of fragmentation) embryos are chromosomally abnormal [31]. In addition, fragments positioned in the cleavage cavity may cause distortion of division planes leading to abnormal compaction, cavitation, and blastocyst formation [27].

With the exception of fragmentation, all other parameters of embryo quality were comparable for the population of implanted and not-implanted advanced blastocysts. No differences between the two groups were observed for the distribution in different categories of number of blastomeres on day 2 and on day 3, compaction rate and cleavage pattern. However, it should be taken into account that in case of elective single advanced blastocyst transfer, embryologists preferentially opt to transfer embryos having 4 cells on day 2 and 8 cells on day 3.

Also we attempted to investigate whether advanced blastocysts with the ideal cleavage pattern (4 cells on day 2 to 8 cells on day 3) had an increased implantation potential compared to advanced blastocysts of the same quality with other cleavage patterns. Blastocysts with an ideal cleavage pattern (4 cells on day 2 to 8 cells on day 3) seemed not to have increased implantation potential compared with blastocysts with another pattern of division. However, an embryo with cleavage pattern 4 cells-8 cells might be more likely to become an advanced blastocyst compared with other cleavage patterns. The above findings suggest that, at the advanced blastocyst stage, the embryo has already reached a high developmental status, and that the morphological parameters of the advanced blastocysts on day-5 itself is of prior importance, rather than its previous cleavage pattern.

Since the implantation rate was as high as 45.8%, it may be assumed that the selection of the embryo for transfer on day 5 was carried out correctly. It should not be overlooked, however, that embryo quality is not the only factor related to success in IVF/ICSI cycles. Once a high-quality blastocyst has been selected, it seems that other than embryological characteristics further determine the implantation potential of the selected blastocyst (female age, endometrial receptivity, previous patient history). [32,33].

## Conclusion

The current study has highlighted that once a patient has high-quality blastocysts, the transfer of an advanced blastocyst may result in implantation in almost half of the patients. The only embryological parameters that seemed to have an effect on the implantation ability in this study were the developmental stage on day-5 and the fragmentation rate on day-3. Which morphological parameter is of prior importance to improve the implantation potential in day 5 embryo transfer needs to be further investigated.

## Competing interests

Authors declare the complete absence of any competing interest (personal or financial) with other people or organizations.

## Authors' contributions

TdR carried out embryological analysis and drafted the manuscript. GV carried out embryological analysis, study design, and revised the manuscript. EGP carried out statistical analysis, study design, and wrote the manuscript. LVL carried out embryological analysis, study design, and revised the manuscript. PD and AVS critically revised the manuscript.
